# A Brief Mindfulness-Based Family Psychoeducation Intervention for Chinese Young Adults With First Episode Psychosis: A Study Protocol

**DOI:** 10.3389/fpsyg.2019.00516

**Published:** 2019-03-11

**Authors:** Herman Hay-Ming Lo, Wing-Chung Ho, Elsa Ngar-Sze Lau, Chun-Wai Lo, Winnie W. S. Mak, Siu-Man Ng, Samuel Yeung-Shan Wong, Jessica Oi-Yin Wong, Simon S. Y. Lui, Cola Siu-Lin Lo, Edmund Chiu-Lun Lin, Man-Fai Poon, Kong Choi, Cressida Wai-Ching Leung

**Affiliations:** ^1^Department of Applied Social Sciences, Faculty of Health and Social Sciences, The Hong Kong Polytechnic University, Kowloon, Hong Kong; ^2^Department of Social and Behavioural Sciences, The City University of Hong Kong, Kowloon, Hong Kong; ^3^Department of Special Education and Counselling, The Education University of Hong Kong, Tai Po, Hong Kong; ^4^Specialist in Psychiatry, Private Practice, Hong Kong, Hong Kong; ^5^Department of Psychology, The Chinese University of Hong Kong, Shatin, China; ^6^Department of Social Work and Social Administration, The University of Hong Kong, Pokfulam, Hong Kong; ^7^The Jockey Club School of Public Health and Primary Care, The Chinese University of Hong Kong, Shatin, Hong Kong; ^8^Department of Psychiatry, Castle Peak Hospital, Hong Kong, Hong Kong; ^9^Integrative Community Centre for Mental Wellness, Baptist Oi Kwan Social Service, Hong Kong, Hong Kong; ^10^Integrative Community Centre for Mental Wellness, Richmond Fellowship of Hong Kong, Hong Kong, Hong Kong; ^11^The Society of Rehabilitation and Crime Prevention, Hong Kong, Hong Kong

**Keywords:** mindfulness-based intervention, family psychoeducation, psychosis, mixed methods, randomized controlled clinical study

## Abstract

Family psychoeducation (FPE) has been recommended as a major component in the treatment of psychosis. Many previous studies have implemented an intensive program design that often only emphasized improvements in patients’ illness outcomes but the benefits for caregivers were limited. There have been calls for a time-limited but cost-effective FPE program to mitigate the looming reality of the suffering of people with psychosis and their families. A Brief Mindfulness-Based Family Psychoeducation for psychosis program is developed to reduce caregivers’ burden and promote young adult’s recovery. A randomized controlled trial will be conducted to compare this intervention with an ordinary FPE intervention. Both arms will involve six sessions, with a total contact time of 12 h. 300 caregivers of young adults who have experienced first episode psychosis within last 3 years will be recruited. Program effectiveness will be assessed by comparing outcomes measuring the caregivers’ burden, mental health symptoms, positive well-being, and the young adult’s mental health symptoms during the study and at 9-month post-randomization. The role of expressed emotions, interpersonal mindfulness, and non-attachment in mediating these outcomes will be explored. An additional qualitative approach Photovoice is selected to explore the complex family experiences and the benefits of mindfulness from the caregivers’ personal perspectives.

**Trial Registration:** The trial is registered with the United States Clinical Trials Registry (ClinicalTrials.gov): NCT03688009.

## Introduction

### Psychosis and Its Impact on Young Adults and Families

Psychosis is defined as a mental, behavioral, or emotional disorder that has been medically diagnosed for at least 1 year. Psychosis usually results in serious functional impairment, which substantially interferes with or limits one or more major life activities and functions in social, family, and vocational/educational contexts (Lefley, 2009). The lifetime prevalence of psychosis is around 0.7 to 2.5% of the general population (Kessler et al., 1994; Chang et al., 2017).

Psychosis has a marked increase in prevalence between the ages of 15 and 17. The majority of psychosis manifests between ages 20 and 30, with a median age for first psychotic episode of 22 (Kessler et al., 2007). Young adults with psychosis often have restricted social networks and experience great social isolation (Bebbington and Kuipers, 2008). One study reported that at their first contact with psychiatric service, over 40% of young adults were not in school or employment (Marwaha et al., 2007). A meta-analysis reported that 34.5% of individuals with psychosis perpetrated violent behaviors before their admission to psychiatric services (Large and Nielssen, 2011). Recent studies have found that 42% of patients with first-episode psychosis reported suicidal ideation, and 9.4% committed violent behaviors (Chang et al., 2014, 2015). Young adults with psychosis experience a high-risk period that places immense strain and anxiety on family caregivers.

### Family Caregivers of Young Adults With Psychosis

Family caregiving is defined as one’s commitment to the welfare of another family member, and the provision of voluntary care to meet their physical, psychological, and developmental needs (Revenson et al., 2016). Family caregivers often take up their role without any formal preparation, knowledge, resources, or skills, and frequently experience great psychological burden. Such burden can be assessed in empirical terms as the consequences for the family’s physical and psychological well-being. Managing family members’ bizarre behaviors, fluctuating emotions, suicidal ideation, and unemployment after the onset of psychosis are the major sources of caregiving burden (Wong et al., 2012). Burden can also be perceived in subjective terms by individual caregivers, relating to their negative emotions arising from the suffering of the family member, such as loss and grief, and the negative perceptions of relatives and community members (Lefley, 2009).

Some studies of caregiving have focused on family expressed emotions (EEs), a robust predictor of relapses and overall outcome of psychosis, including number of relapses, hospitalization, and symptom severity (Hooley, 2007; Weintraub et al., 2017). High EEs are defined as criticism, hostility, and over-involvement (Brown et al., 1972), and are considered a reciprocal process in family interactions that are developed and increased after the onset of illness, particularly in the first 5 years (Hooley, 2007).

However, other studies of EEs targeting psychosis have suggested a more complicated picture. A recent review of higher EEs concluded that higher levels of criticism predicted positive symptoms of psychosis, but the association between negative symptoms of psychosis and high EEs was weak (Cechnicki et al., 2013). Further, avoidant coping, negative appraisals of the illness’s impact, and perceived losses were associated more frequently with family EEs. Among families facing psychosis, over-involvement is often normal, as young adults have not fully developed their own self-care abilities, and the boundaries between positive concern and family over-involvement are blurred (McNab and Linszen, 2009). Higher levels of EEs are more likely to be found in families from non-Western cultures, such as Indian, Japanese, and Chinese families (Bhugra and McKenzie, 2010). Higher EEs may be the cultural norm in these countries, as they coexist with positive factors such as family connectedness and strong family ties.

### Family Psychoeducation for Caregivers

Family psychoeducation (FPE) is a core component of treatment for psychosis, as recommended by the Schizophrenia Patient Outcomes Research Team and the National Institute for Health and Care Excellence (Dixon et al., 2009; National Institute for Health and Clinical Excellence, 2015). Many FPE programs apply cognitive behavioral models with an emphasis on modifying family dysfunction, characterized by high EEs, and usually involve the teaching of practical knowledge and skills required to manage psychosis (Sellwood et al., 2007; McFarlane, 2016). Other program components include empathic understanding, social support, normalization of reactions, resource information, exchange of coping strategies, and installation of hope (Lefley, 2009).

The efficacy of FPE varies across studies and there is room for improvement. An earlier meta-analysis reported that FPE largely benefited people with psychosis. The 1-year relapse rate for the treatment group was 6 to 12% while those for the control group was 41 to 53% (Falloon, 2004). A recent systematic review reported variations in benefits of FPE in psychological distress of caregivers. The overall quality of study was low, and limitations including high heterogeneity and small sample sizes were identified (Yesufu-Udechuku et al., 2015).

To improve the efficacy of FPE, first, its design should be more theory-driven. A recent review study concluded that it remains unclear how and why FPE works (Gracio et al., 2016). The assumptions about higher EEs and their role in preventing relapses have not been investigated in studies of FPE, and many highly burdened families have not shown higher EEs (Lefley, 2009). EE and more family related variables as potential mediating variables should be included in studies of FPE.

Second, FPE interventions should be simple, practical, effective and sustainable. Previous program designs have been relatively long and unstructured. For example, a study of an 18 session FPE program reported selected improvements in the functioning of patients and families and caregiving burden, and fewer relapses (Chien and Wong, 2007). However, such an intensive program will create difficulties in implementation, and families were burdened by participating in such an intensive program (Glynn, 2012).

Third, developmental needs and cultural issues for psychosis should be included as a guiding theoretical model (McNab and Linszen, 2009). The management of young adults with psychosis is beyond the comprehension of most parents, particularly from Chinese or East Asian societies, who often pre-occupied about their obligation to raise, or support a healthy and successful child, and experience strong sense of loss, guilt, and frustration for not being able to help the young people to fully resume their premorbid functioning (Wong, 2000). Reduced caregiving burden has been associated with the acceptance of the patient’s behaviors, illness course and caregiver’s own social functioning (Magliano et al., 2005). Therefore in a FPE, it might be beneficial to promote the quality of non-attachment in caregiving, which is defined by an absence of holding, or a fixation on desirable values or behaviors as determined (Sahdra and Shaver, 2013). Besides, based on a new conception of recovery for psychosis, families can be strengthened in finding hope and commitment through building new self-identities for young adults, and being involved in developing meaningful goals and strengths, beyond the label of mental patients and achieving symptom control and medication compliance (Davidson et al., 2005).

### Mindfulness-Based Intervention and Its Application in Families

Mindfulness-based interventions (MBIs) have been widely adopted as an evidence-based approach in supporting people with chronic medical conditions (Bohlmeijer et al., 2010). Mindfulness is defined as paying attention, non-judgmentally, to the present moment (Kabat-Zinn, 2013). In MBI, instructors provide guided training on mindfulness exercises, including body scan, stretching, and mindful sitting. An inquiry into participant needs is followed by an exploration of their personal experiences. New insights and understandings about participants’ reactions to stress are addressed.

Mindfulness-based intervention originates from a stress and coping model for individuals with chronic conditions (Kabat-Zinn, 2013; Segal et al., 2013). It can improve their attention, promote tolerance of unpleasant sensations and feelings, and facilitate cognitive changes and effective coping, and all these benefits may be helpful in supporting the caregivers in managing the caregiving burden and EE. A recent review suggests the mindfulness role of adaptive emotion regulation that MBI can reduce intensity of emotional distress, enhance emotional recovery, reduced negative self-referential processing, and promote the engagement in goal-directed behaviors (Roemer et al., 2015). In dynamic bi-directional relationships in caregiving, family caregivers experience stress arising from monitoring psychotic symptoms and alerting the young people with psychosis who has impaired insights about their own care needs (McNab and Linszen, 2009). Moreover, the entire family often experiences the spill-over effect of the psychosis, as the original functioning of all other members are frequently disrupted, and caregivers may feel overwhelmed in their anxieties and diminished abilities to cope (Quah, 2015).

Some studies have applied MBI to parents or caregivers and suggested it can benefit participant’s family systems. 86 parents of children with mixed psychiatric diagnoses were recruited in a non-randomized clinical trial of a MBI. Improvements were found not only in the mental health symptoms of both the children and their parents, but also in parenting behaviors, and parental stress (Bögels et al., 2014). This study included outcomes of caregivers, care recipients, and quality in caregiving but two major limitations were lacking randomization and the heterogeneity of the sample. A study of 40 wives of people with schizophrenia reported that compared with those in control group receiving no intervention MBP participants had a higher level of resilience, but other clinical outcomes of the caregivers and people with psychosis were not included (Solati, 2017). More studies were based on parents or caregivers mixed medical conditions using MBI. For example, 141 caregivers of persons with chronic conditions were randomized into mindfulness-based stress reduction program or self-help control group. Participants reported reduction in depressive and anxiety symptoms, self-efficacy and mindfulness (Hou et al., 2014). Although such rigorous study give support to the effectiveness of MBI, outcomes of the care recipients are not included in the study design and we cannot be certain whether MBI can benefit the family members who did not participate in the intervention directly.

A brief mindfulness-based family psychoeducation (MBFPE) for first episode psychosis is developed and a randomized controlled trial of the MBFPE with an active control group will be conducted. The MBFPE will be offered to caregivers only but outcomes of young people with psychosis will also be investigated. Mindfulness is consistent with a holistic view of recovery. Caregivers may learn to appreciate and incorporate the key recovery principles of recovery such as self-determination, resilience, respect, and hope (Davis and Kurzban, 2012; Murray et al., 2017) and positive indicators of recovery will be included as one of the outcome measures in this study.

As the theoretical underpinning for applying mindfulness in family caregiving is emerging, there is a call for studies of MBI that specified clear targets of intervention that can investigate and generate knowledge about mechanisms of change (Dimidjian and Segal, 2015). This study may contribute additional evidences of applying MBI in families as well as the change mechanism of caregiving process. The mediating effects of interpersonal mindfulness, EE, and non-attachment will be explored: first, inspired by on the positive outcome on interpersonal mindfulness in some recent studies (Lo et al., in press), we shall test whether caregivers’ mindfulness can be improved after MBI and whether it will mediate the changes of other outcomes. Second, as studies have selected emotion regulation as a change mechanism of mindfulness (Gratz and Tull, 2010; Roemer et al., 2015), we explore if MBI can reduce EEs that can lead to reduction of caregiver burden, other positive and negative mental health indicators and improvements in overall family functioning. Finally, caregivers may have potential benefits in cognitive flexibility in MBI. In view of the mediating effect of over-attachment in the change of anxiety and depression in MBI (Lo et al., 2013), we investigate the role of non-attachment in the outcomes of MBFPE in this study (Sahdra et al., 2010; Jansen et al., 2015, 2017).

### Objectives

The current study will test the following three hypotheses based on the literature:

(i) Caregivers who participate in a MBFPE program will experience less caregiving burden, less anxiety and depressive symptoms, less physical distress, more positive caregiving experiences, higher levels of interpersonal mindfulness, higher levels of well-being, higher levels of perceived family functioning, higher levels of non-attachment, and less unplanned medical consultations than FPE participants.
(ii) Young adults will report reduced psychiatric symptoms, higher levels of recovery, lower EEs, and less days in hospitalization after their caregivers’ participation in MBFPE than FPE.
(iii) Improvements in interpersonal mindfulness, EE, and non-attachment will mediate improvements in caregiving burden and other outcomes in caregivers and adults with psychosis.

The embedded use of Photovoice in the intervention will add knowledge about experience of caregivers for further improvements in FPE and MBI.

## Materials and Equipment

### Study Design

This study will be a multi-site randomized controlled trial, with a mixed-methods design. The effects of the intervention will be tested using a two-arm randomized controlled trial, comparing the MBFPE (arm 1) to an ordinary FPE (arm 2). Assessments will be made before the intervention (T1), after the intervention (T2), and at 9-month follow-up (T3). The program effects will be tested using both between-subject differences (comparison of the two arms) and within-subject differences (comparison of measures at T1, T2, and T3).

Both MBFPE and FPE include 1 h psychoeducation video that has been standardized for implementation. Another 1 h is used for mindfulness training in arm 1 and for sharing and discussion in arm 2. The strengths of this study is the use of an active control group in arm 2. If hypotheses 1 and 2 are supported, it will offer a strong evidence that a brief 6-h mindfulness training can produce additional effects to ordinary psychoeducation programs.

### Participants

#### Sample Size Estimation

**FIGURE 1:**
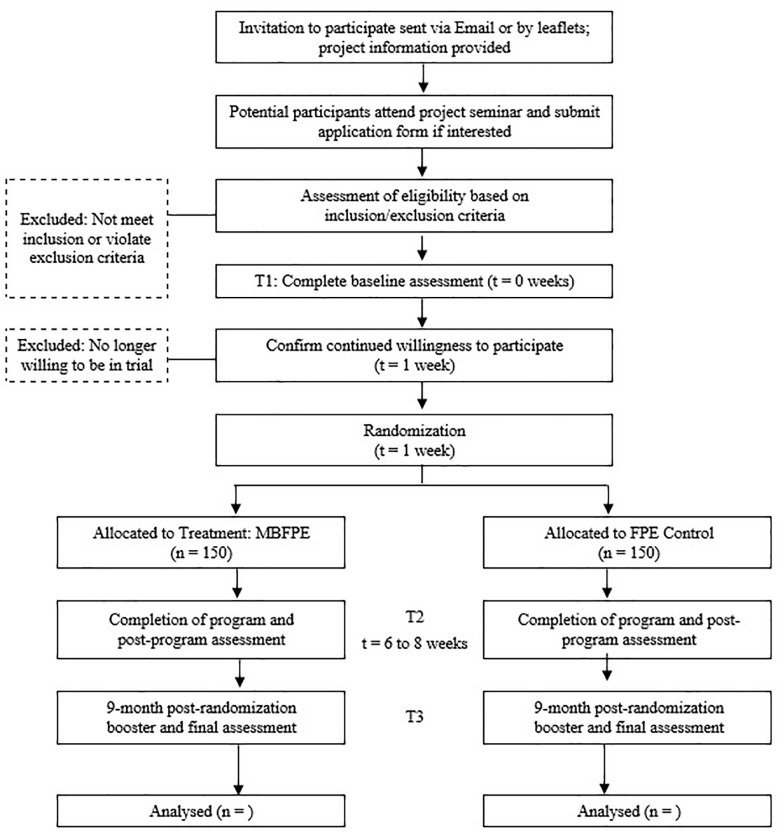
Flew diagram of participant allocation.

As no similar study has been conducted, the sample size calculation is based on a study of a MBI for parents of children with developmental disabilities, in which an effect size of 0.65 in stress (Lo et al., 2017), with an estimation of an effect size of 0.15 for arm 2. For a two-tailed α error of 5%, an 80% power, and a test of two independent groups, the required sample size will be 128 participants for two arms (Cohen, 1988). We further adjust the sample size based on an estimation of drop-out rate and intra-class correlation. The estimated drop-out rate of 15% is based on two local studies of MBIs (Hou et al., 2014; Lo et al., 2017). The estimated intra-class correlation of 0.07 is based on the first author’s two recent mindfulness multi-site studies, in which the intra-class correlation ranged from 0 to 0.07, and related studies in Western contexts (Adams et al., 2004; Lo et al., 2017). 300 caregivers will be recruited for this study. A flowchart of the study’s planned recruitment and implementation is illustrated in Figure 1.

#### Recruitment Process

The study will be based on convenience sampling, as it is not possible to collect a full list of young adults with psychosis and caregivers due to confidentiality of medical records and personal data. The study inclusion criteria are as follows: (1) caregivers of a young adult under the age of 35 who has experienced first episode psychosis in the last 3 years. Psychosis include Schizophrenia Spectrum and other psychotic disorders and bipolar and related disorders in Diagnostic and Statistical Manual of Mental Disorders, 5th Edn (DSM-5; American Psychiatric Association, 2013); (2) caregivers who have offered care to the young adult for at least 1 year; (3) young people with the capacity to provide informed consent and to respond the questions in assessment interviews. The exclusion criteria are as follows: (1) caregivers who have been diagnosed with psychosis or developmental disabilities, such as intellectual disabilities, which may cause difficulties in comprehending the content of the program; (2) caregivers or young adults who refuse to participate in regular psychiatric consultations.

The research project “Family Psychoeducation for Psychosis” will be announced and promoted in all psychiatric units of the Hospital Authority, by psychiatrists in private practice, local school social work and youth mental health services, and student counseling services in all tertiary education institutions via emails and mailed project leaflets. Three integrated mental health service NGOs and one mental health hospital will participate in the study by assisting with its promotion, recruitment, program implementation, and data collection. The service centers and the University of the Principal Investigator are located at the north-western, middle, and eastern districts with convenient public transportation and all caregivers living in the city can reach one of these sites within 1 h.

All interested caregivers will be invited to participate in a briefing session, in which the rationale of the study and components of FPE will be explained. Both arms will be referred to as a “Family Psychoeducation Program,” and the term “mindfulness” will not be used for arm 1, to minimize the potential placebo effect. After they indicate their intentions to participate in the study, written consent forms will be distributed and collected from the caregiver. During the briefing session, the research team will explain the invitation of young adults with psychosis in the study so that they may choose to prepare their family members about the arrangement. A research team member will initiate follow-up contacts with the young adults with psychosis and invite them to participate in this study. It will be emphasized that the participation of study is independent from the mental health care he received from collaborators, if suitable, and they have the rights to withdraw from the study at any time without negative consequences. Priority will be given to caregivers if their young adults with psychosis agree to participate in the assessment. Social workers from the NGO collaborators will provide standard care to the selected young adults, and encourage them to participate in the study. Research assistants will administer the random assignment using computer generated programming, and treatment allocation will be blinded to the participants. The participants will be randomly assigned to the MBFPE (arm 1), or ordinary FPE (arm 2) conditions. However, to ensure the use of blinding in the project, all collaborators who are involved in promotion and recruitment and the assessors of young people outcomes will not be involved in the randomization and program implementation.

In order to improve the attrition rate and the intention to participate the study at all three time points, cash remuneration coupons of HKD100 (about USD12) will be provided to caregivers at T2 and T3. HKD200 (about USD25) coupon will be provided to young adults who complete the study at T1, T2, and T3. Offering cash remuneration coupon is an incentive to research participants and is commonly adopted as a strategy to enhance recruitment and enable them to participate in the study without financial sacrifice (Grady, 2005). The underlying ethical concerns are researchers and collaborators should not influence the participants or force people to participate in the studies with coercion through the use of excessive incentive (Largent and Lynch, 2017). All researcher team members including collaborators should be clear about the research ethics that participants are free to participate and to withdraw from the study at any time without any negative consequences. In reality, a significant proportion of the caregivers and young adults are homemakers, unemployed, or students with no or limited income. Cash coupons should be offered to offset their cost to participate in the study (VanderWalde and Kurzban, 2011). The amount of cash coupon are set within nominal standard according the living standard in the city that the study is conducted.

### Measures

**FIGURE 2:**
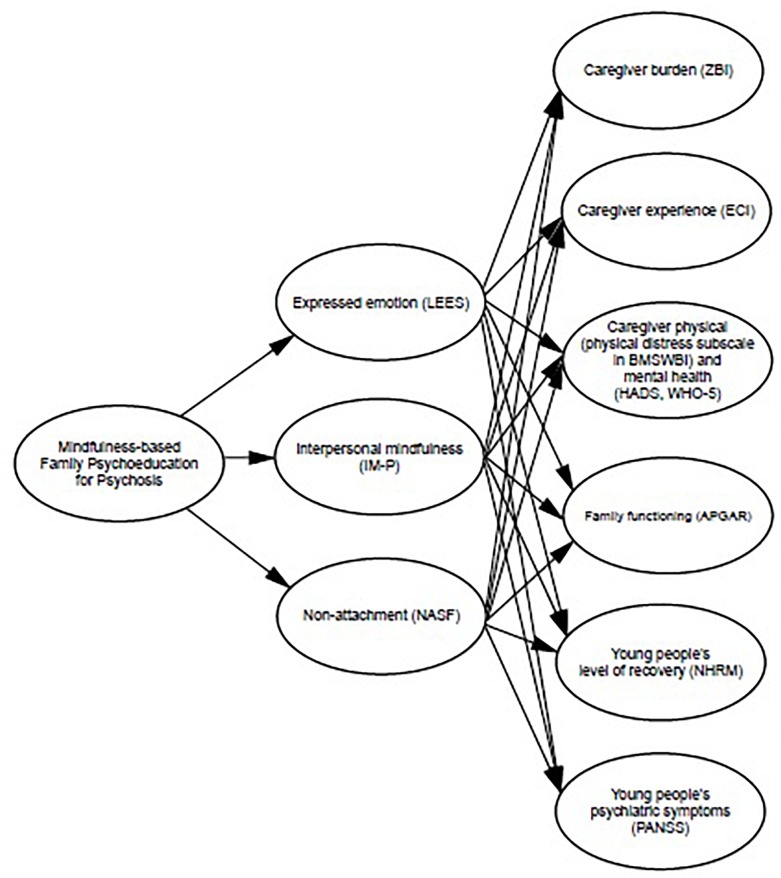
Mediation model.

All proposed outcomes, mediators and measures are illustrated in Figure 2.

#### Primary Outcome Variable

##### Caregivers’ burden

The Zarit Burden Interview is a 22-item measure of caregivers’ perceived stress level (ZBI; Zarit et al., 1980). The degree of burden is measured across areas including health, psychological well-being, finances, social life, and relationship with the patient. Sample items include “Do you feel that because of the time you spend with your relative, you don’t have time for yourself?” and “Do you feel that you have lost control of your life since your relative’s illness?” The caregivers will be asked to indicate the level of discomfort surrounding this question by choosing an answer ranging from 0 “not at all” to 4 “extremely.” The total score range is from 0 to 88. The scale has been validated among caregivers of patients with schizophrenia in Chinese with a high internal consistency (Cronbach’s alpha) of 0.88 (Tang et al., 2017).

#### Secondary Outcome Variables

##### Caregiving experiences

The Experience of Caregiving Inventory (ECI; Szmukler et al., 1996) will be used to measure the caregiving experiences. Three subscales are selected in relation to the purposes of this study: stigma (e.g., “feeling unable to tell anyone of the illness,” five items), effects on the family (e.g., “How his/her illness affects special family events,” seven items), and positive experience in caregiving (e.g., “I have discovered strengths in myself,” 14 items). The stigma score can range from 0 (no experience of stigma) to 20 (strong experience of stigma). The effects on the family score can range from 0 (no negative effects on the family) to 28 (strong negative effects on the family). The positive experience score can range from 0 (no positive experience in caregiving) to 56 (strong positive experience in caregiving). ECI has been validated among Chinese caregivers with internal consistencies from 0.75 to 0.85 for selected subscales (Lau and Pang, 2007).

##### Caregivers’ physical health

The 14-item physical distress subscale in the Body-Mind-Spirit Well-Being Inventory (BMSWBI; Ng et al., 2005) will be used to measure caregivers’ physical health. It includes symptoms of physical distress such as headache, chest pain, and fatigue. Participants rate their level of physical distress in the past week, from 0 “no distress at all” to 10 “extreme distress.” The total score for physical health can range from 0 (no distress) to 140 (high distress). The physical distress subscale of BMSWBI showed a high internal consistency of 0.87 (Ng et al., 2005).

##### Caregivers’ mental health symptoms

The Hospital Anxiety and Depression Scale is selected to measure the caregivers’ mental health symptoms (HADS; Zigmond and Snaith, 1983). Caregivers rate their symptoms from 0 “low” to 4 “severe,” and the anxiety and depression symptoms score can range from 0 to 21. HADS was validated and the internal consistencies for anxiety and depression subscales were 0.77 and 0.82 respectively (Leung et al., 1999).

##### Caregivers’ well-being

The WHO-5 Well-Being Index (Johansen, 1998) is a well-validated measure of psychological well-being. It includes five positive well-being statements (e.g., “my daily life has been filled with things that interest me”). Caregivers will be asked to indicate their degree of well-being in the past 2 weeks, from 0 “at no time” to 5 “all of the time.” The total score can range from 0 to 25, with higher scores indicating a better subjective perception of well-being. The WHO-5 has been validated in a Chinese sample with a high internal consistency of 0.86 (Kong et al., 2016).

##### Caregivers’ perceived family functioning

The Family APGAR Scale (Smilkstein et al., 1982) is a validated 5-item measure of perceived family functioning, with five dimensions of family functioning including adaptation, partnership, growth, affection, and resolve. A sample item is “I am satisfied with the way my family expresses affection and responds to my emotions, such as anger, sorrow, and love.” The caregivers are invited to rate their perceived family functioning from 0 “hardly ever” to 2 “almost always.” The total score can range from 0 to 10, with higher scores indicating better perceived family functioning. The Family APGAR has been widely adopted in Chinese samples and a high internal consistency of 0.91 was reported in a recent study (Nan et al., 2013).

##### Caregivers’ interpersonal mindfulness

The Interpersonal Mindfulness in Parenting Scale is a measure of interpersonal mindfulness for parents (IM-P; Duncan, 2007). The Chinese version of IM-P is well-validated with four subscales including compassion for child (seven items), emotional awareness in parenting (six items), non-judgmental acceptance in parenting (six items) and listening with full awareness (four items) (Lo et al., 2018). Samples items include “I try to be understanding and patient with my family member when he/she is having a hard time” and “When my family member does something that upsets me, I try to keep my emotions in balance.” The total score can range from 23 to 115, with higher scores indicating a higher level of interpersonal mindfulness in caregiving. The overall internal consistencies of Chinese version of IM-P was 0.85 and those of four subscales are 0.70 and 0.84 (Lo et al., 2018).

##### Caregivers’ non-attachment

The Non-Attachment Scale was developed to measure a general state of being psychologically and socially adaptive (Sahdra et al., 2010). The Chinese short form of the Non-Attachment Scale (NAS-SF) has eight items, and caregivers will be asked to rate each item from 1 “strongly disagree” to 6 “strongly agree” (Chio et al., 2018). A sample item is “I can accept the flow of events in my life without hanging onto them or pushing them away.” The total score can range from 8 to 48, with higher scores indicating a higher level of non-attachment, and a high internal consistency of 0.91 was reported (Chio et al., 2018).

##### Young adult’s level of recovery

The Mental Health Recovery Measure (Young and Bullock, 2005) is a 30-item measure of young adults’ mental health recovery relating to their experience in psychosis. It provides a comprehensive evaluation of the recovery experience from a young adult’s perspective, without measuring psychiatric symptoms. The items cover positive dimensions in recovery including overcoming stuckness, self-empowerment, learning and self-redefinition, basic functioning, overall well-being, new potential, advocacy/enrichment, and spirituality. Each item records the young adult’s level of agreement on a five-point scale (1 = totally disagree to 5 = totally agree). Sample items include “The way I think about things helps me to achieve my goals” and “Even though I may still have problems, I value myself as a person of worth.” The total score can range from 30 to 150, with higher scores indicating a higher level of recovery, and a high internal consistency of 0.93 was reported in a Chinese scale validation study (Ye et al., 2013).

##### Young adult’s family expressed emotions

The Level of Expressed Emotion Scale (LEES; Cole and Kazarian, 1988) is a validated 12-item measure of family EEs. This measure is based on self-reports from young adults with psychosis, with subscales in criticism (four items), hostility (four items), and over-involvement (four items). Sample items include “My family members often accuse me of making things up when I’m not feeling well” and “My family members insist on knowing where I’m going.” The youths will be asked to rate each item on a five-point scale (1 = totally disagree to 4 = totally agree). Criticism, hostility and over-involvement sub-scores can range from 4 (low EE) to 16 (high EE). The three subscale scores are summed to compute a total score for family EEs. A recent Chinese study of LEES reported participants over cut-off points showed a 6.3 times elevated 12-month relapse rate compared with the counterparts (Ng et al., 2019). The internal consistency of the whole LEES was 0.84 and the three subscales were 0.75 to 0.77 (Ng and Sun, 2011).

##### Young adult’s psychiatric symptoms

The Positive and Negative Syndrome Scale is a measure of psychiatric symptoms (PANSS; Kay et al., 1987; Chen et al., 2005). An independent research assistant with at least 3 years of mental health practice experience will rate the young adult’ scores after a clinical interview. The scale includes seven items for positive symptoms (e.g., delusions), seven items for negative symptoms (e.g., blunted affect), and 14 items for general psychopathology (e.g., lack of judgment and insight). The positive and negative symptom subscale scores can range from 7 (less severe) to 49 (very severe). The general psychopathology subscale score can range from 16 (less severe) to 112 (very severe). The internal consistencies of PANSS based on Chinese samples were 0.73 to 0.84 (Chan et al., 2004).

##### Other behavioral indicators

Caregivers’ unplanned medical consultations and young adults’ days spent hospitalized will be recorded.

### Stepwise Procedures

#### Program Planning and Training

The themes and content of arms 1 and 2 are summarized in Table 1. For MBFPE (arm 1), 1-h mindfulness training is infused with 1-h FPE. For ordinary FPE (arm 2), the entire session is reserved for knowledge and skills about managing psychosis, and for mutual support. Both programs include understanding psychosis, medication, treatment management, mental health service collaboration, attention to caregivers’ experiences and distress, strategies for improving communication and problem-solving, and crisis planning, based on best practices for working with psychosis (Froggatt et al., 2007; McNab and Linszen, 2009). Both arms will involve six sessions, with a total contact time of 12 h. Arm 1 includes 10 min of daily mindfulness homework. The research team has produced a psychoeducation video that covers these key topics. It includes mini-lectures by multi-disciplinary professionals including a psychiatrist, a clinical psychologist, two psychiatric nurses, an occupational therapist, several social workers, and sharing from caregivers and peer support workers who are young adults in recovery and have been involved in community education. The video will be supplemented by discussion and sharing for participants. Protocols have been developed and refined based on feedback from instructors, participants, and NGO social workers in the pilot study.

**Table 1:** Proposed intervention program outline: content of mindfulness-based family psychoeducation (MBFPE) (arm 1) and family psychoeducation (FPE) (arm 2).

Session themes	Mindfulness-based family psychoeducation (MBFBE) (arm 1)	Family psychoeducation (FPE) (arm 2)

**Core process**	**Non-judgmental, collaborative inquiry, self-care**	**Knowledge sharing, problem-solving, mutual support**
(1) Understanding the impact of caregiving stress	(a) Orientation to the program (b) Mindfulness practice: mindful eating, body scan (c) Video: caregiver’s reaction of onset of SMI (d) Discussion: awareness of the impact of caregiving on body and mind (e) Homework: body scan	(a) Orientation to the program (b) Sharing and discussion: stress and reactivity in caregiving (c) Video: caregiver’s reaction of onset of SMI (d) Discussion: normalizing the reactions of caregiver stress

(2) The impact of psychosis to young adults	(a) Mindfulness exercises: mindful stretching, mindful walking (b) Inquiry: mindfulness exercises (c) Video show: understanding positive and negative symptoms (d) Homework: mindful stretching, 3 min breathing, and photovoice (a pleasant moment)	(a) Sharing and discussion: issues in handling symptoms and behaviors of family member in recovery (b) Video show: understanding positive and negative symptoms (c) Discussion: strategies on symptom management and promoting recovery

(3) The experience of young adults with psychosis in recovery	(a) Mindfulness exercises: mindful sitting, mindful communication (b) Inquiry: mindfulness exercises and photovoice (c) Video show: sharing of persons in recovery (d) Homework: mindful sitting, 3 min breathing, and photovoice (an unpleasant moment)	(a) Sharing and discussion: goals and needs for holistic recovery (b) Video show: sharing of persons in recovery (c) Discussion on understanding and communicating with family members in recovery

(4) The struggles of caregivers	(a) Mindfulness exercises: mindfulness with difficult moments, mindful communication (b) Inquiry: mindfulness exercises and photovoice (c) Video show: challenges in caregiving and self- care (d) Homework: mindfulness with difficult moments, 3-min breathing, and photovoice (my family)	(a) Sharing and discussion: stress and coping in caregiving, and difficulties in communicating with family members with SMI (b) Video show: challenges in caregiving and self-care (c) Discussion on preventing compassion fatigue

(5) The partnership with multi-disciplinary team in recovery	(a) Mindfulness exercise: be-friending (b) Inquiry: mindfulness exercise and photovoice (c) Video show: understanding treatment and services for adults with SMI (d) Homework: be-friending, 3 min breathing, and photovoice (recovery)	(a) Sharing and discussion: experiences and issues about working with mental health professionals (b) Video show: understanding treatment and services for adults with SMI (c) Discussion on strategies for promoting recovery and partnership with professionals

(6) Review of learning	(a) Mindfulness exercises: body scan, mindful sitting (b) Inquiry: mindfulness exercises and photovoice (c) Video show: relapse plan and management (d) Review: what I learn in the program	(a) Sharing and discussion: risk and relapse management (b) Video show: relapse plan and management (c) Review: what I learn in the program

(7) 9-month post-randomization booster	Review: changes and benefits in mindfulness Inquiry: photovoice	Review: changes and benefits of the program

Instructors for arm 1 will require basic professional training in MBI, plus regular personal mindfulness practice and at least 2 years of experience in conducting mindfulness-based programs. Instructors for arm 2 will be mental health professionals with practical experience working with psychosis for over 2 years.

#### Implementation and Quantitative Data Collection

After the first assessment (T1), caregivers who meet the inclusion criteria will be randomized into an MBFPE (arm 1) or an ordinary FPE (arm 2). After the intervention, participants in both arms will complete the second assessment (T2). Both arms will be delivered in group format, with 12 to 18 caregivers in each group. Programs will be conducted at NGO service centers or in the psychiatric unit of a public hospital. At 9-month post-randomization (T3) will be offered as a booster and final assessment for both arms.

#### Intervention Fidelity

To ensure intervention fidelity, all program sessions will be audio-recorded and an independent rater will listen to 20% of the clips (randomly selected) and assess whether each element in the intervention protocol has been implemented consistently. All raters shared same qualifications of the instructors. Higher concordance rates will signify greater fidelity to the intervention protocol, which will be carefully monitored throughout the study. The treatment fidelity of arm 1 will be further assessed using the Mindfulness-based Interventions-Teaching Assessment Criteria Scale (Crane et al., 2012).

#### Qualitative Data Collection

The embedded mixed-methods design to be used will examine the program outcomes through experimental design and explore the intervention process using the qualitative study method Photovoice. The quantitative data will be used to investigate the outcomes and effectiveness of the MBFPE, and to test whether it can attain positive changes for family caregivers and young adults with psychosis. A supplementary, qualitative, participatory action research method called Photovoice will be adopted to engage the participants to contribute to more candid and in-depth knowledge of the caregiving process, and to explore the “processes” occurring during MBFPE and the follow-up period (Wang, 1999). In the qualitative study, caregivers will contribute to offering a unique contextual understanding of the outcomes, and ideas, insights, suggestions, and questions that have not been adequately addressed in the literature. This will also ensure the internal validity of the intervention (Bryman, 2006).

Photovoice, as qualitative method technique, can facilitate people to record and reflect about their strengths and concerns of about being a caregiver, to foster dialog about the caregiving process and personal experiences with MBPFE, by sharing ideas and discussions about their photos (Wang, 1999; Ho et al., 2011). The procedures include the following: (1) in MBFPE sessions 2 to 5, the Photovoice themes will be included as homework assignments; (2) guidelines will be offered at the end of the sessions and participants will be encouraged to take pictures using their smartphones; (3) participants will write down their reflections on the images and share them in the following sessions, and send their pictures and reflections to the research team; (4) in subsequent sessions, time will be allocated for collaborative enquiry on the pictures and reflections. With the participants’ consent, the pictures, reflections, and content of the in-session enquiries will be displayed. All participants will be involved in sharing and commenting on the pictures and reflections, in terms of both mindfulness and caregiving. (5) At T3, all pictures will be re-displayed and the participants will be invited to view their pictures, share additional reflections about their caregiving experience and participation in MBFPE, and highlight their reflections on MBPFE and caregiving.

## Anticipated Results

### Quantitative Data Analysis

#### Intervention Effects

All analyses will be carried out according to the intent-to-treat approach (Moher et al., 2010). Missing values will be handled with multiple imputation procedure (Sterne et al., 2009). MANOVA will be used to evaluate the effects of the MBFPE (arm 1), relative to the FPE (arm 2), and the primary and secondary outcome measures will be analyzed. In addition to the immediate program effects, the outcomes measured at T2 and T3 will be compared, to assess whether maintenance effects are sustained at 9-month post-randomization.

Priority will be given to caregivers if their young people with psychosis agree to participate in the assessment. It is expected that at least half of the young people will participate in the study. The participation and attrition rates of the young adults will be monitored and analyzed at three time-points. Analysis will be conducted to compare the differences in outcomes between the group with and without young people’s participation in terms of their background profile. If any significant difference is detected, implications and limitations in interpretation of the findings will be provided.

#### Qualitative Data Analysis

The author and the research team will apply grounded theory to analyze the Photovoice images, participants’ reflections, and MBFPE transcripts (Padgett, 2008). Conceptual categories will arise through the data interpretation. The process will encourage the research team to be reflexive about the prior interpretive frames, interests and research context, relationships with participants, and modes of generating and recording empirical materials in the process of analyses (Charmaz, 2006). The team will watch the videotapes of the MBFPE sessions, and study the transcripts of themes, categories, and concepts generated during the Photovoice inquiries. The research team will share these reflections with the MBFPE instructors, and invite the participants to clarify, elaborate upon, and critique the interpretations. Using constant comparative method, the researcher is able to do what is necessary to develop a theory, through categorizing, coding, delineating categories and connecting them. The cycle of comparison and reflection on ‘old’ and ‘new’ material can be repeated several times (Boeije, 2002). The first MBFPE session will be coded, followed by the second session, then the coding of the two sessions will be compared. The coding will be added to or altered throughout the study. Theoretical sampling will also be undertaken, to fill gaps in the analyses (Belgrave, 2014).

Collaboration with community stakeholders and the democratization of knowledge construction are strategies for enhancing research credibility for participatory action research (Balazs and Morello-Frosch, 2013). Through the analyses of *Photovoice* that will be contributed by caregivers, the research team members produce ideas of what and how mindfulness has been useful to caregivers to community stakeholders and share responsibility for the advancement of knowledge. Knowledge is constructed and improved by an open, collaborative workspace, and is democratized among caregivers, people with psychosis, mindfulness instructors, mental health professionals, and researchers (Scardamalia, 2002). Social workers from the collaborating NGOs have been involved with the team since the pilot study, helping formulate the Photovoice procedure. The participants will contribute to the study by sharing their personal reflections during MBFPE, based on their pictures. During this process, the most salient features of these dialogs will be jointly determined by the instructor and the participants. Transcripts will be recorded and themes identified by the researchers. At T3, all pictures and the preliminary analysis will be discussed with all participants until concurrence on the coding and interpretations is reached between the researchers and participants. The participants will then be able to comment on the analysis findings. Further meetings between the research team members, and additional Photovoice sharing sessions for the mental health professionals will help to strengthen the reliability of the qualitative study findings and conclude the data analysis process.

## Discussion

Family caregivers play a pivotal role in treatment and recovery of psychosis, as most people have their onset of illness in young adulthood and continue to live with their families. They often take up the caregiving burden without adequate knowledge and support and studies have shown that over one-third of them experienced significant emotional distress such as depression (Chen et al., 2016). FPE has been reported to have positive outcomes, but many limitations have been identified, such as long duration and intensive design, and emphasis on benefits for patients but not caregivers. There have been calls for a time-limited but more cost-effective FPE program to mitigate their hardship under the looming realities of people with psychosis and their families.

We have developed a brief MBFPE program, to reduce caregivers’ burden and promote young adult’s recovery. In this study, we will conduct a randomized controlled trial of MBFPE to investigate the effects after intervention and at 9-month post-randomization using multiple outcome measures for both caregivers and young adults in recovery. The study will include multiple sites and 300 family caregivers will be randomly allocated to MBFPE or FPE. Successful completion of the study and confirmation of the hypotheses will contribute to the evidence on the effectiveness of MBFPE and MBIs. The low intensity of the intervention will provide a sustainable treatment option for policymakers, service providers, family caregivers, and other stakeholders. MBFPE may also be considered for common mental disorders such as major depressive disorder and obsessive compulsive disorder, as the caregivers of people with these mental health issues often suffer comparable stress levels (Renshaw et al., 2005; van Wijngaarden et al., 2009).

An additional qualitative approach Photovoice is selected to explore the complex family experiences and the benefits of mindfulness from the caregivers’ personal perspectives. Caregivers can offer their voices about their burdens and how mindfulness can benefit families, through their involvement in a photo taking activity during the psychoeducation program. In this study, the data collection and analyses of Photovoice are embedded in the intervention. Further studies may explore the application of Photovoice or image-making activities to understand the impact of MBI.

We predict a major difficulty in the recruitment of young people with psychosis in this study. Some of them may refuse to participate in the study at the first stage and more may dropout in the follow-up period. Analysis will be conducted to compare the differences in outcomes between the group with and without young people’s participation in terms of young people’s profiles. If any significant difference is detected, implications in interpretation of findings and further studies will be discussed in study report and peer-reviewed publications. On the other hand, research team members and collaborators should be cautious about the mental health status of the young adults with psychosis and explain to them about the meaning of cash remuneration coupon with clarity. Under all circumstances they are free to participate and to withdraw from the study at any time with negative consequences.

## Ethics Statement

Ethical approval for this study has been obtained from the Human Subjects Ethics Sub-Committee of The Hong Kong Polytechnic University (Reference No. HSEARS20161122002). The research team will explain all information about the study in a briefing session and an information sheet. All participants will be asked to sign on a written consent form.

## Author Contributions

HL designed the project, obtained the project funding, developed the intervention materials, refined the study protocol, and takes responsibility for the overall coordination of the project. W-CH and EN-SL contributed to designing the qualitative study and will perform the Photovoice data analyses. C-WL will provide consultation with the research team and caregivers, based on his expertise in psychiatry. WM contributed to the study methods and scale measurements. S-MN contributed to the study methods, based on his expertise in EE studies. SW contributed to the program design and will offer medical advice to the instructors and caregivers. JW, SL, CS-LL, and EC-LL contributed to improving the study design and producing psychoeducation video, recruiting, and implementing the study. MP and KC contributed to improving the protocol, producing the psychoeducation video, recruiting, and implementing the study. CW-CL contributed to producing the psychoeducation video, recruiting, and implementing the study.

## Conflict of Interest Statement

The authors declare that the research was conducted in the absence of any commercial or financial relationships that could be construed as a potential conflict of interest.
